# 3-Diethyl­amino-6-[(*Z*)-(4-hy­droxy­anilino)methyl­idene]cyclo­hexa-2,4-dienone

**DOI:** 10.1107/S1600536810045939

**Published:** 2010-11-13

**Authors:** Erkan Soydemir, Orhan Büyükgüngör, Çiğdem Albayrak

**Affiliations:** a Department of Physics, Ondokuz Mayıs University, TR-55139 Samsun, Turkey; b Sinop Faculty of Education, Sinop University, Sinop, Turkey

## Abstract

In the mol­ecule of the title compound, C_17_H_20_N_2_O_2_, the aromatic rings are oriented at a dihedral angle of 6.23 (22)°. Intra­molecular N—H⋯O hydrogen bonding involving the amine H atom and the carbonyl O atom affects the conformation of the mol­ecule. One of the ethyl arms is disordered over two conformations, with occupancies of 0.59 (2) and 0.41 (2). The crystal packing is stabilized by inter­molecular C—H⋯O and O—H⋯O hydrogen bonds, and weak C—H⋯π inter­actions.

## Related literature

For general background to Schiff bases, see: Odabaşoğlu *et al.* (2004 ▶); Hadjoudis *et al.* (1987 ▶); Hodnett & Dunn (1970 ▶); Misra *et al.* (1981 ▶); Agarwal *et al.* (1983 ▶); Varma *et al.* (1986 ▶); Singh & Dash (1988 ▶); Pandey *et al.* (1999 ▶); El-Masry *et al.* (2000 ▶); Samadhiya & Halve (2001 ▶); Xu *et al.* (1994 ▶); Calligaris *et al.* (1972 ▶); Cohen *et al.* (1964 ▶); Moustakali-Mavridis *et al.* (1978 ▶). For related strucures: Ersanlı *et al.* (2003 ▶); Şahin *et al.* (2005 ▶).
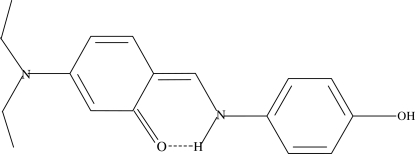


## Experimental

### 

#### Crystal data

C_17_H_20_N_2_O_2_
*M* *_r_* = 284.35
Monoclinic, 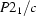

*a* = 8.2648 (4) Å
*b* = 11.8968 (4) Å
*c* = 16.6149 (7) Å
β = 112.400 (3)°
*V* = 1510.39 (11) Å^3^
*Z* = 4
Mo *K*α radiation
μ = 0.08 mm^−1^
*T* = 296 K
0.54 × 0.44 × 0.26 mm

#### Data collection

Stoe IPDS 2 diffractometer
Absorption correction: integration (*X-RED32*; Stoe & Cie, 2002 ▶) *T*_min_ = 0.957, *T*_max_ = 0.979
18967 measured reflections
3143 independent reflections
2416 reflections with *I* > 2σ(*I*)
*R*_int_ = 0.067

#### Refinement

*R*[*F* ^2^ > 2σ(*F* ^2^)] = 0.065
*wR*(*F* ^2^) = 0.189
*S* = 1.06
3143 reflections
208 parameters
13 restraints
H atoms treated by a mixture of independent and constrained refinement
Δρ_max_ = 0.37 e Å^−3^
Δρ_min_ = −0.27 e Å^−3^

### 

Data collection: *X-AREA* (Stoe & Cie, 2002 ▶); cell refinement: *X-AREA*; data reduction: *X-RED32* (Stoe & Cie, 2002 ▶); program(s) used to solve structure: *SHELXS97* (Sheldrick, 2008 ▶); program(s) used to refine structure: *SHELXL97* (Sheldrick, 2008 ▶); molecular graphics: *ORTEP-3 for Windows* (Farrugia, 1997 ▶); software used to prepare material for publication: *WinGX* (Farrugia, 1999 ▶).

## Supplementary Material

Crystal structure: contains datablocks I, global. DOI: 10.1107/S1600536810045939/si2307sup1.cif

Structure factors: contains datablocks I. DOI: 10.1107/S1600536810045939/si2307Isup2.hkl

Additional supplementary materials:  crystallographic information; 3D view; checkCIF report

## Figures and Tables

**Table 1:** Hydrogen-bond geometry (Å, °) *Cg*1 is the centroid of the C8–C13 ring.

*D*—H⋯*A*	*D*—H	H⋯*A*	*D*⋯*A*	*D*—H⋯*A*
N1—H1*B*⋯O2	0.90 (3)	1.80 (3)	2.574 (2)	142 (2)
O1—H1*A*⋯O2^i^	0.87 (4)	1.75 (4)	2.599 (2)	165 (4)
C16*A*—H16*A*⋯O2^ii^	0.96	2.52	3.306 (7)	139
C5—H6⋯*Cg*1^iii^	0.93	2.93	3.799 (3)	156

Symmetry codes: (i) 
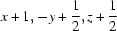
; (ii) 
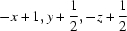
; (iii) 
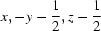
.

**Fig. 1.**
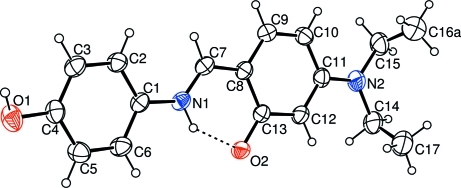
An ORTEP view of (I), with the atom-numbering scheme and 30% probability displacement ellipsoids. Only the major disorder component C16A are displayed. The dashed line indicates the intramolecular hydrogen bond.

**Fig. 2.**
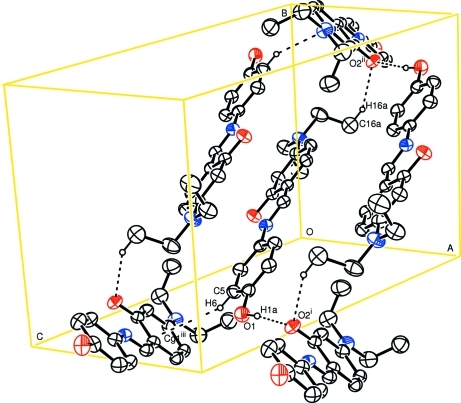
A packing diagram for (I), hydrogen bonds and C—H···π interaction are drawn as dashed lines. [Symmetry codes: (i) 1 - x,1/2 + y, 1/2 + z; (ii) x + 1,1/2 - y, 1/2 - z; (iii) x,1/2 - y,1/2 + z]

## supplementary crystallographic information

### Comment

Schiff bases are used as substrates in the preparation of number of industrial and biologically active compounds *via* ring closure, cycloaddition and replacement reactions.Some Schiff base derivatives are also known to have biological activities such as antimicrobial (El-Masry *et al.*, 2000; Pandey *et al.*, 1999); antifungal (Singh & Dash 1988; Varma *et al.*, 1986); antitumor (Hodnett & Dunn 1970; Misra *et al.*, 1981; Agarwal *et al.*, 1983) and as herbicides (Samadhiya & Halve, 2001). In additin, Schiff bases have been used widely as ligands in the field of coordinatin chemistry (Calligaris *et al.*, 1972). There are two caracteri properties of Schiff bases, *viz*. photochromism and thermochromism (Cohen *et al.*, 1964; Moustakali-Mavridis *et al.*, 1978). These properties result from proton transfer from the O atom to the imin N atom (Hadjoudis *et al.*, 1987; Xu *et al.*, 1994).

In the molecule of (I), the C13=O2 and N1—C7 bond lengths of 1.301 (2) and 1.307 (3) Å, respectively are in good agreement with those observed in 2-[(2-hydroxy-4-nitrophenyl)aminomethylene]cyclohexa-3,5-dien-1(2*H*)-one [1.298 (2) and 1.308 (2) Å; Ersanlı *et al.*, 2003] and 2-Hydroxy-6-[(2-methoxyphenyl)aminomethylene]cyclohexa-2,4-dienone [1.2931 (17) and 1.3043 (19) Å; Şahin *et al.*, 2005]. The study of Schiff bases has led to the proposal that molecules exhibiting thermochromism are planar, while those exhibiting photochromism are non-planar. This planarity of the molecule allows the proton to be transferred through the hydrogen bond in the ground state with a small energy requirement (Odabaşoǧlu *et al.*, 2004; Hadjoudis *et al.*, 1987). The dihedral angle between benzene rings A(C1—C6) and B(C8—C13) is 6.49 (22)° (Fig.1). These two rings are twisted slightly about the methylene amino group with torsion angles of -0.5 (3)° [C2—C1—N1—C7] and 179.48 (18)° [N1—C7—C8—C9], respectively. C16 was disordered over two positions A and B (C16A and C16B) wiht the occupancy factors refined to 0.59 (2) and 0.41 (2). Intramolecular N1—H1A···O2 hydrogen bonding contributes to the overall planarity of the molecule. In crystal packing, the weak [C5—H6···*Cg*1(*x*,- 1/2 - *y*, - 1/2 + *z*)] interaction, and the hydrogen bonds [C16A—H16A···O2(1 - *x*,1/2 + *y*,1/2 - *z*)] and [O1—H1A···O2(1 + *x*,1/2 - *y*,1/2 + *z*)] are listed in Table 1 and labelled in Fig.2.

### Experimental

The compound (*Z*)-6-[(4-hydroxyphenylamino)methylene]-3 -(diethylamino)cyclohexa-2,4-dienone was prepared by refluxing a mixture of a solution containing 5-(diethylamino)-2-hydroxybenzaldehyde (0.5 g, 2.59 mmol) in 20 ml e thanol and a solution containing 4-hydroxyaniline (0.28 g, 2.59 mmol) in 20 ml e thanol. The reaction mixture was stirred for 1 h under reflux. The crystals of the title compound were obtained by slow evaporation from ethyl alcohol (yield % 73; m.p. 468–470 K).

### Refinement

All H atoms were placed in calculated positions except H1A and H1B which were located in a difference fourier map. All carbon-bound H atoms were refined using a riding model with C—H = 0.93 to 0.97 Å and *U*_iso_(H) = 1.2–1.5 *U*_eq_ (C).

### Crystal data

**Table:**

C_17_H_20_N_2_O_2_	*F*(000) = 608
*M**_r_* = 284.35	*D*_x_ = 1.250 Mg m^−^^3^
Monoclinic, *P*2_1_/*c*	Mo *K*α radiation, λ = 0.71073 Å
Hall symbol: -P 2ybc	Cell parameters from 18967 reflections
*a* = 8.2648 (4) Å	θ = 1.7–28.0°
*b* = 11.8968 (4) Å	µ = 0.08 mm^−^^1^
*c* = 16.6149 (7) Å	*T* = 296 K
β = 112.400 (3)°	Prism, brown
*V* = 1510.39 (11) Å^3^	0.54 × 0.44 × 0.26 mm
*Z* = 4	

### Data collection

**Table:**

Stoe IPDS 2 diffractometer	3143 independent reflections
Radiation source: fine-focus sealed tube	2416 reflections with *I* > 2σ(*I*)
graphite	*R*_int_ = 0.067
Detector resolution: 6.67 pixels mm^-1^	θ_max_ = 26.5°, θ_min_ = 2.2°
rotation method scans	*h* = −10→10
Absorption correction: integration (*X-RED32*; Stoe & Cie, 2002)	*k* = −14→14
*T*_min_ = 0.957, *T*_max_ = 0.979	*l* = −20→20
18967 measured reflections	

### Refinement

**Table:**

Refinement on *F*^2^	Primary atom site location: structure-invariant direct methods
Least-squares matrix: full	Secondary atom site location: difference Fourier map
*R*[*F*^2^ > 2σ(*F*^2^)] = 0.065	Hydrogen site location: inferred from neighbouring sites
*wR*(*F*^2^) = 0.189	H atoms treated by a mixture of independent and constrained refinement
*S* = 1.06	*w* = 1/[σ^2^(*F*_o_^2^) + (0.0969*P*)^2^ + 0.3716*P*] where *P* = (*F*_o_^2^ + 2*F*_c_^2^)/3
3143 reflections	(Δ/σ)_max_ < 0.001
208 parameters	Δρ_max_ = 0.37 e Å^−^^3^
13 restraints	Δρ_min_ = −0.27 e Å^−^^3^

### Special details

**Table:**

Geometry. All e.s.d.'s (except the e.s.d. in the dihedral angle between two l.s. planes) are estimated using the full covariance matrix. The cell e.s.d.'s are taken into account individually in the estimation of e.s.d.'s in distances, angles and torsion angles; correlations between e.s.d.'s in cell parameters are only used when they are defined by crystal symmetry. An approximate (isotropic) treatment of cell e.s.d.'s is used for estimating e.s.d.'s involving l.s. planes.
Refinement. Refinement of *F*^2^ against ALL reflections. The weighted *R*-factor *wR* and goodness of fit *S* are based on *F*^2^, conventional *R*-factors *R* are based on *F*, with *F* set to zero for negative *F*^2^. The threshold expression of *F*^2^ > σ(*F*^2^) is used only for calculating *R*-factors(gt) *etc*. and is not relevant to the choice of reflections for refinement. *R*-factors based on *F*^2^ are statistically about twice as large as those based on *F*, and *R*- factors based on ALL data will be even larger.

### Fractional atomic coordinates and isotropic or equivalent isotropic displacement parameters (Å^2^)

**Table:**

	*x*	*y*	*z*	*U*_iso_*/*U*_eq_	Occ. (<1)
C1	0.8028 (2)	0.31443 (16)	0.65717 (13)	0.0483 (5)	
C2	0.9660 (3)	0.35845 (19)	0.70654 (14)	0.0549 (5)	
H3	1.0126	0.4163	0.6844	0.066*	
C3	1.0587 (3)	0.31624 (19)	0.78840 (14)	0.0581 (5)	
H2	1.1680	0.3463	0.8212	0.070*	
C4	0.9927 (3)	0.22989 (19)	0.82301 (13)	0.0550 (5)	
C5	0.8278 (3)	0.1876 (2)	0.77383 (16)	0.0627 (6)	
H6	0.7800	0.1309	0.7963	0.075*	
C6	0.7352 (3)	0.22939 (19)	0.69203 (15)	0.0593 (6)	
H5	0.6253	0.2001	0.6595	0.071*	
C7	0.7361 (2)	0.42738 (17)	0.52483 (13)	0.0501 (5)	
H7	0.8386	0.4689	0.5495	0.060*	
C8	0.6253 (2)	0.44989 (16)	0.43902 (12)	0.0470 (5)	
C9	0.6675 (3)	0.53469 (18)	0.39085 (14)	0.0536 (5)	
H9	0.7688	0.5766	0.4180	0.064*	
C10	0.5666 (3)	0.55735 (19)	0.30686 (14)	0.0567 (5)	
H10	0.6000	0.6134	0.2774	0.068*	
C11	0.4090 (3)	0.49573 (19)	0.26311 (13)	0.0556 (5)	
C12	0.3648 (3)	0.41193 (19)	0.30976 (13)	0.0552 (5)	
H12	0.2638	0.3701	0.2816	0.066*	
C13	0.4654 (2)	0.38791 (17)	0.39687 (12)	0.0473 (5)	
C14	0.1382 (4)	0.4570 (2)	0.13497 (16)	0.0802 (8)	
H15A	0.0600	0.5023	0.0875	0.096*	
H15B	0.0819	0.4447	0.1759	0.096*	
C15	0.3476 (4)	0.6042 (3)	0.12686 (17)	0.0855 (8)	
H14A	0.4063	0.6671	0.1634	0.103*	
H14B	0.2416	0.6319	0.0815	0.103*	
C16A	0.4682 (17)	0.5521 (6)	0.0854 (7)	0.096 (3)	0.59 (2)
H16A	0.4960	0.6078	0.0508	0.144*	0.59 (2)
H16B	0.4097	0.4900	0.0491	0.144*	0.59 (2)
H16C	0.5741	0.5262	0.1304	0.144*	0.59 (2)
C16B	0.388 (3)	0.5630 (9)	0.0567 (10)	0.097 (4)	0.41 (2)
H16D	0.4141	0.6249	0.0267	0.145*	0.41 (2)
H16E	0.2888	0.5224	0.0171	0.145*	0.41 (2)
H16F	0.4868	0.5137	0.0785	0.145*	0.41 (2)
C17	0.1651 (5)	0.3472 (3)	0.1001 (2)	0.1033 (11)	
H17A	0.0543	0.3102	0.0725	0.155*	
H17B	0.2406	0.3013	0.1468	0.155*	
H17C	0.2177	0.3588	0.0583	0.155*	
N1	0.7013 (2)	0.35026 (14)	0.57193 (11)	0.0500 (4)	
H1B	0.597 (3)	0.316 (2)	0.5437 (17)	0.066 (7)*	
N2	0.3028 (3)	0.51988 (19)	0.17918 (12)	0.0712 (6)	
O1	1.0820 (2)	0.18478 (17)	0.90278 (11)	0.0723 (5)	
H1A	1.193 (5)	0.197 (3)	0.919 (3)	0.131 (14)*	
O2	0.41712 (18)	0.31201 (13)	0.43958 (9)	0.0573 (4)	

### Atomic displacement parameters (Å^2^)

**Table:**

	*U*^11^	*U*^22^	*U*^33^	*U*^12^	*U*^13^	*U*^23^
C1	0.0409 (9)	0.0490 (11)	0.0465 (10)	0.0055 (8)	0.0073 (8)	−0.0005 (8)
C2	0.0445 (10)	0.0592 (12)	0.0530 (12)	−0.0014 (9)	0.0097 (9)	0.0059 (10)
C3	0.0429 (10)	0.0648 (13)	0.0548 (12)	0.0007 (9)	0.0053 (9)	−0.0002 (10)
C4	0.0488 (10)	0.0650 (13)	0.0471 (11)	0.0128 (9)	0.0135 (9)	0.0064 (9)
C5	0.0546 (11)	0.0642 (14)	0.0655 (14)	0.0015 (10)	0.0185 (11)	0.0146 (11)
C6	0.0444 (10)	0.0603 (13)	0.0619 (13)	−0.0036 (9)	0.0076 (9)	0.0058 (10)
C7	0.0429 (9)	0.0485 (10)	0.0525 (11)	0.0005 (8)	0.0109 (8)	−0.0046 (9)
C8	0.0449 (9)	0.0468 (10)	0.0456 (10)	0.0028 (8)	0.0129 (8)	−0.0024 (8)
C9	0.0497 (10)	0.0532 (11)	0.0560 (12)	−0.0039 (9)	0.0178 (9)	−0.0026 (9)
C10	0.0634 (12)	0.0543 (12)	0.0568 (12)	−0.0037 (10)	0.0279 (10)	0.0028 (10)
C11	0.0632 (12)	0.0575 (12)	0.0430 (10)	0.0009 (10)	0.0167 (9)	0.0002 (9)
C12	0.0554 (11)	0.0583 (12)	0.0428 (10)	−0.0078 (9)	0.0086 (9)	−0.0013 (9)
C13	0.0471 (10)	0.0473 (10)	0.0433 (10)	0.0010 (8)	0.0125 (8)	−0.0015 (8)
C14	0.0868 (16)	0.0904 (19)	0.0479 (13)	−0.0054 (12)	0.0081 (12)	0.0082 (12)
C15	0.119 (2)	0.0810 (18)	0.0530 (14)	−0.0084 (16)	0.0290 (15)	0.0098 (13)
C16A	0.097 (3)	0.096 (3)	0.096 (3)	−0.0016 (10)	0.0374 (14)	0.0005 (10)
C16B	0.097 (4)	0.097 (4)	0.096 (4)	−0.0010 (10)	0.0381 (18)	0.0011 (10)
C17	0.138 (3)	0.095 (2)	0.0714 (19)	−0.017 (2)	0.0339 (19)	−0.0090 (17)
N1	0.0400 (8)	0.0524 (10)	0.0467 (9)	−0.0004 (7)	0.0044 (7)	−0.0006 (7)
N2	0.0859 (13)	0.0756 (13)	0.0445 (10)	−0.0067 (10)	0.0161 (9)	0.0084 (9)
O1	0.0591 (10)	0.0981 (14)	0.0537 (9)	0.0155 (9)	0.0147 (8)	0.0213 (9)
O2	0.0523 (8)	0.0624 (9)	0.0452 (8)	−0.0123 (6)	0.0050 (6)	0.0069 (7)

### Geometric parameters (Å, °)

**Table:**

C1—C6	1.385 (3)	C12—C13	1.397 (3)
C1—C2	1.387 (3)	C12—H12	0.9300
C1—N1	1.409 (3)	C13—O2	1.301 (2)
C2—C3	1.377 (3)	C14—N2	1.479 (3)
C2—H3	0.9300	C14—C17	1.481 (4)
C3—C4	1.387 (3)	C14—H15A	0.9700
C3—H2	0.9300	C14—H15B	0.9700
C4—O1	1.358 (3)	C15—C16B	1.415 (9)
C4—C5	1.389 (3)	C15—N2	1.465 (3)
C5—C6	1.374 (3)	C15—C16A	1.541 (8)
C5—H6	0.9300	C15—H14A	0.9700
C6—H5	0.9300	C15—H14B	0.9700
C7—N1	1.307 (3)	C16A—H16A	0.9600
C7—C8	1.396 (3)	C16A—H16B	0.9600
C7—H7	0.9300	C16A—H16C	0.9600
C8—C9	1.412 (3)	C16B—H16D	0.9600
C8—C13	1.441 (3)	C16B—H16E	0.9600
C9—C10	1.352 (3)	C16B—H16F	0.9600
C9—H9	0.9300	C17—H17A	0.9600
C10—C11	1.429 (3)	C17—H17B	0.9600
C10—H10	0.9300	C17—H17C	0.9600
C11—N2	1.366 (3)	N1—H1B	0.90 (3)
C11—C12	1.394 (3)	O1—H1A	0.87 (4)
			
C6—C1—C2	119.00 (19)	N2—C14—H15A	108.9
C6—C1—N1	117.45 (17)	C17—C14—H15A	108.9
C2—C1—N1	123.54 (19)	N2—C14—H15B	108.9
C3—C2—C1	119.8 (2)	C17—C14—H15B	108.9
C3—C2—H3	120.1	H15A—C14—H15B	107.8
C1—C2—H3	120.1	C16B—C15—N2	116.3 (5)
C2—C3—C4	121.39 (19)	N2—C15—C16A	110.1 (4)
C2—C3—H2	119.3	C16B—C15—H14A	124.5
C4—C3—H2	119.3	N2—C15—H14A	109.6
O1—C4—C3	123.0 (2)	C16A—C15—H14A	109.6
O1—C4—C5	118.5 (2)	C16B—C15—H14B	84.4
C3—C4—C5	118.50 (19)	N2—C15—H14B	109.6
C6—C5—C4	120.2 (2)	C16A—C15—H14B	109.6
C6—C5—H6	119.9	H14A—C15—H14B	108.2
C4—C5—H6	119.9	C15—C16A—H16A	109.5
C5—C6—C1	121.10 (19)	C15—C16A—H16B	109.5
C5—C6—H5	119.4	H16A—C16A—H16B	109.5
C1—C6—H5	119.4	C15—C16A—H16C	109.5
N1—C7—C8	122.57 (18)	H16A—C16A—H16C	109.5
N1—C7—H7	118.7	H16B—C16A—H16C	109.5
C8—C7—H7	118.7	C15—C16B—H16D	109.5
C7—C8—C9	120.58 (18)	C15—C16B—H16E	109.5
C7—C8—C13	121.43 (18)	H16D—C16B—H16E	109.5
C9—C8—C13	118.00 (17)	C15—C16B—H16F	109.5
C10—C9—C8	122.60 (19)	H16D—C16B—H16F	109.5
C10—C9—H9	118.7	H16E—C16B—H16F	109.5
C8—C9—H9	118.7	C14—C17—H17A	109.5
C9—C10—C11	120.5 (2)	C14—C17—H17B	109.5
C9—C10—H10	119.8	H17A—C17—H17B	109.5
C11—C10—H10	119.8	C14—C17—H17C	109.5
N2—C11—C12	121.0 (2)	H17A—C17—H17C	109.5
N2—C11—C10	121.2 (2)	H17B—C17—H17C	109.5
C12—C11—C10	117.73 (19)	C7—N1—C1	129.19 (18)
C11—C12—C13	122.95 (19)	C7—N1—H1B	112.7 (16)
C11—C12—H12	118.5	C1—N1—H1B	118.1 (16)
C13—C12—H12	118.5	C11—N2—C15	122.7 (2)
O2—C13—C12	121.47 (18)	C11—N2—C14	120.8 (2)
O2—C13—C8	120.34 (17)	C15—N2—C14	116.4 (2)
C12—C13—C8	118.20 (19)	C4—O1—H1A	111 (3)
N2—C14—C17	113.2 (3)		
			
C6—C1—C2—C3	0.9 (3)	C11—C12—C13—O2	177.4 (2)
N1—C1—C2—C3	−178.06 (19)	C11—C12—C13—C8	−2.5 (3)
C1—C2—C3—C4	0.1 (3)	C7—C8—C13—O2	2.8 (3)
C2—C3—C4—O1	178.7 (2)	C9—C8—C13—O2	−177.25 (18)
C2—C3—C4—C5	−1.3 (3)	C7—C8—C13—C12	−177.39 (19)
O1—C4—C5—C6	−178.5 (2)	C9—C8—C13—C12	2.6 (3)
C3—C4—C5—C6	1.5 (3)	C8—C7—N1—C1	176.75 (18)
C4—C5—C6—C1	−0.5 (4)	C6—C1—N1—C7	−179.4 (2)
C2—C1—C6—C5	−0.7 (3)	C2—C1—N1—C7	−0.5 (3)
N1—C1—C6—C5	178.3 (2)	C12—C11—N2—C15	−177.6 (2)
N1—C7—C8—C9	179.48 (18)	C10—C11—N2—C15	4.1 (4)
N1—C7—C8—C13	−0.5 (3)	C12—C11—N2—C14	−0.5 (4)
C7—C8—C9—C10	178.19 (19)	C10—C11—N2—C14	−178.9 (2)
C13—C8—C9—C10	−1.8 (3)	C16B—C15—N2—C11	110.8 (10)
C8—C9—C10—C11	0.7 (3)	C16A—C15—N2—C11	83.7 (6)
C9—C10—C11—N2	177.9 (2)	C16B—C15—N2—C14	−66.3 (10)
C9—C10—C11—C12	−0.4 (3)	C16A—C15—N2—C14	−93.5 (6)
N2—C11—C12—C13	−177.0 (2)	C17—C14—N2—C11	−81.1 (3)
C10—C11—C12—C13	1.4 (3)	C17—C14—N2—C15	96.1 (3)

### Hydrogen-bond geometry (Å, °)

**Table:**

Cg1 is the centroid of the C8–C13 ring.

**Table:**

*D*—H···*A*	*D*—H	H···*A*	*D*···*A*	*D*—H···*A*
N1—H1B···O2	0.90 (3)	1.80 (3)	2.574 (2)	142 (2)
O1—H1A···O2^i^	0.87 (4)	1.75 (4)	2.599 (2)	165 (4)
C16A—H16A···O2^ii^	0.96	2.52	3.306 (7)	139
C5—H6···Cg1^iii^	0.93	2.93	3.799 (3)	156

Symmetry codes: (i) *x*+1, −*y*+1/2, *z*+1/2; (ii) −*x*+1, *y*+1/2, −*z*+1/2; (iii) *x*, −*y*−1/2, *z*−1/2.
